# Is Ghrelin Synthesized in the Central Nervous System?

**DOI:** 10.3390/ijms18030638

**Published:** 2017-03-15

**Authors:** Agustina Cabral, Eduardo J. López Soto, Jacques Epelbaum, Mario Perelló

**Affiliations:** 1 Laboratory of Neurophysiology, Multidisciplinary Institute of Cell Biology (IMBICE), Universidad de La Plata-Consejo Nacional de Investigaciones Científicas y Técnicas, CONICET, and Comision de Investigaciones de la Provincia de Buenos Aires (CIC), Buenos Aires 1900, Argentina; agustinacabral177@hotmail.com; 2 Department of Neuroscience, Brown University, Providence, RI 02912, USA; ejlopezsoto@gmail.com; 3 Centre Psychiatrie & Neurosciences UMR_S894 INSERM Université Paris Descartes, 75014 Paris, France; jacques.epelbaum@inserm.fr; 4 MECADEV UMR 7179 Centre National de la Recherche Scientifique, Muséum National d’Histoire Naturelle France, 91800 Brunoy, France

**Keywords:** brain, neuron, acyl-ghrelin

## Abstract

Ghrelin is an octanoylated peptide that acts via its specific receptor, the growth hormone secretagogue receptor type 1a (GHSR-1a), and regulates a vast variety of physiological functions. It is well established that ghrelin is predominantly synthesized by a distinct population of endocrine cells located within the gastric oxyntic mucosa. In addition, some studies have reported that ghrelin could also be synthesized in some brain regions, such as the hypothalamus. However, evidences of neuronal production of ghrelin have been inconsistent and, as a consequence, it is still as a matter of debate if ghrelin can be centrally produced. Here, we provide a comprehensive review and discussion of the data supporting, or not, the notion that the mammalian central nervous system can synthetize ghrelin. We conclude that no irrefutable and reproducible evidence exists supporting the notion that ghrelin is synthetized, at physiologically relevant levels, in the central nervous system of adult mammals.

## 1. Introduction

Ghrelin is an octanoylated peptide and the only known natural agonist of a specific G protein-coupled receptor named growth hormone secretagogue receptor type 1a (GHSR-1a). Ghrelin is predominantly synthetized by closed-type enteroendocrine ghrelin cells located within the gastric oxyntic mucosa [1]. Plasma ghrelin concentrations display a surge before meals, decline after meals, and then increase gradually until the next preprandial peak [2]. In addition, plasma ghrelin levels are elevated in energy deficit conditions, such as fasting, malnutrition, anorexia nervosa or cachexia, among others [3,4,5,6]. Ghrelin actions largely occur via GHSR-1a activation in rodent models; however, ghrelin may also act on other unidentified receptors in some tissues and under some conditions [7,8]. GHSR-1a is mainly expressed in the pituitary as well as in some specific areas of the central nervous system, where ghrelin signaling regulates a vast variety of physiological functions [9,10]. In particular, central actions of ghrelin play a major role in the regulation of the body energy homeostasis since ghrelin regulates a variety of food intake-related behaviors [11]. Ghrelin is the only known orexigenic peptide hormone, and its administration to humans or rodents potently increases food intake [11]. Studies in rodents have shown that ghrelin-induced food intake mainly involves the activation of hypothalamic targets, such as the arcuate nucleus (ARC), which are easily accessible to the plasma hormone [12,13,14]. In addition, some, but not all, studies have suggested that vagal afferents may also be involved in the ghrelin-induced food intake [15,16,17,18]. In addition, ghrelin promotes food intake in human and rodents via the modulation of the rewarding properties of certain foods and the motivation to obtain them [19]. These actions of ghrelin seem to involve several brain regions, such as the mesolimbic pathway, which control hedonic components of eating and other aspects of reward processing [19]. Another pivotal action of central ghrelin signaling is the induction of adiposity, which seems independent of food intake regulation, and occurs via regulation of nutrient partitioning by triggering carbohydrates utilization, reducing fat oxidation, and stimulating lipid synthesis [20]. Moreover, ghrelin administration to healthy humans activates some neuroendocrine axes as it strongly increases growth hormone (GH) as well as prolactin, adrenocorticotropic hormone and cortisol plasma levels [21,22]. The ghrelin-induced activation of the hypothalamic–GH–insulin-like growth factor axis seems to involve the action of the hormone at both hypothalamic neurons and somatotroph pituitary cells [23,24,25]. In contrast, studies in mice indicate that ghrelin-induced activation of the hypothalamic–pituitary–adrenal axis mainly occurs at the hypothalamic level [26,27]. The ghrelin-induced activation of these neuroendocrine axes together to the direct or indirect action of ghrelin on other organs, such as the liver or the pancreas, promotes an increase of plasma glucose levels; however, the mechanisms by which ghrelin controls glucose homeostasis remain poorly understood. In humans, ghrelin regulates some functions that depend on the activity of the autonomic nervous system; for instance, ghrelin increases gastric emptying and decreases blood pressure [28,29,30]. GHSR-1a is expressed in all three components of the dorsal vagal complex, and central administration of ghrelin to rodents recapitulates some of these actions of the hormone [31,32,33]; however, the extent to which these effects of ghrelin depend on its action at central level in humans is currently uncertain. Finally, it is interesting to stress that studies in rodents have shown that ghrelin system also impacts on higher cognitive functions, such as learning and memory, but the implications of these studies in humans are currently unclear [34,35].

It is well established that many of the above referred functions of ghrelin require its specific action on different brain regions. However, ghrelin does not cross the blood–brain barrier in a blood-to-brain direction, and the accessibility of circulating ghrelin to the brain seems to be limited to a few brain areas [12,13,36]. The main targets of circulating ghrelin seem to include circumventricular organs such as the area postrema, where plasma ghrelin can freely diffuse through the fenestrated capillaries, or their surrounding brain areas, as it is the case of the hypothalamic ARC that is located in close apposition to the median eminence (ME) [12,37]. Additionally, circulating ghrelin could reach deep brain areas via the cerebrospinal fluid after crossing the blood–cerebrospinal fluid barrier at either the choroid plexus, a specialized layer of cuboidal ependymal cells that surround a core of capillaries in some brain ventricles, and/or the hypothalamic tanycytes, a specialized layer of bipolar ependymal cells that line the floor of the third ventricle and bridge the cerebrospinal fluid and the capillaries of the ME [38,39]. However, cerebrospinal fluid ghrelin concentrations are ~1000-fold lower than plasma ghrelin levels [40]. Thus, the physiological relevance of the ghrelin responsive neurons located in brain areas in distant locations from circumventricular organs or the ventricular system is unclear.

Some features of the ghrelin system support the notion that central GHSR-1a signaling could be regulated independently of both plasma hormone levels and gastrointestinal function. In this regard, an interesting feature of GHSR-1a is its high constitutive activity that makes it able to signal in a ghrelin-independent manner [41,42]. Such constitutive activity of GHSR-1a has been not only shown to strongly impact on the neuronal activity but also proposed to have some physiological impact [43,44,45]. Additionally, GHSR-1a can heterodimerize with other G protein-coupled receptors, including the melanocortin 3 receptor, the serotonin 2C receptor, the somatostatin 5 receptor and the dopamine D1 and D2 receptors [46,47,48,49,50]. Besides its intrinsic constitutive activity, heterodimerization has been proposed as another mechanism to modulate specific functions of GHSR-1a independently of ghrelin binding. In addition to these mechanisms, the central production of ghrelin has been considered as a simpler mechanism to locally regulate GHSR-1a signaling. Unfortunately, however, and despite almost twenty years of investigations, the evidences for a central production of ghrelin have been inconsistent and, as a consequence, it is still a matter of debate if ghrelin can be brain produced. Here, we attempt to provide an objective compilation of all available data, to the best our knowledge, related to the efforts of different laboratories to find indications of central production of ghrelin (Table 1), and then, we offer a comprehensive discussion of the available data supporting, or not, the possibility that ghrelin can be centrally synthesized.

## 2. Ghrelin Synthesis

Mammalian ghrelin genes display a high percentage of sequence identity giving rise to a well conserved ghrelin peptide [81,82]. Human ghrelin gene expands more than 7 kilobase (kb) in chromosome 3, and consists of two noncoding exons, named exon 1 and exon 0, as well as four coding exons (exon 1 to exon 4) [83]. The exons 1 to 4 encode the ghrelin pre-prohormone polypeptide (pre-proghrelin), in which the signal peptide is encoded in exon 1, and the coding sequence of the ghrelin is encoded by parts of exons 1 and 2. Ghrelin gene expression takes place at high levels in the enteroendocrine ghrelin cells [1], and is tightly regulated in order to ensure ghrelin production according to varying energy requirements [84,85]. Ghrelin gene expression seems to be regulated by an upstream TATA box-like promoter region sequence, where many transcription factors potentially bind [86,87]. In addition, the human ghrelin promoter contains both positive and negative regulatory regions. In this regard, NF-κB has been shown to suppress the ghrelin promoter gene activity, while Nkx2.2 seems to stimulate it [88,89]. Thus, the increase of ghrelin gene expression in fasting conditions correlates with a prompt decrease in *NF-κB* gene expression while *Nkx2.2* mRNA expression remains unaltered [89]. It is interestingly to stress that the ghrelin gene can produce multiple alternative mRNA transcripts [83]. The major bioactive product of the ghrelin gene seems to be ghrelin; however, ghrelin gene displays a complex transcriptional pattern that also generates mRNA transcripts that do not code for ghrelin, but instead for C-terminal region of pre-proghrelin, splice variants that differ in their 5′ untranslated regions and antisense transcripts [83,90,91]. Some of the alternative ghrelin mRNA transcript variants include the in1-ghrelin transcript that retains the intron 1 leading to a truncated form of ghrelin, the ex2-deleted ghrelin transcript that lacks exon 2 and also leads to a truncated form of ghrelin, the ex3-deleted ghrelin transcript that lacks the exon 3 containing the coding region for obestatin, and the in2c-ghrelin transcript that contains the coding region for ghrelin and an intron 2-derived sequence but lacks the obestatin sequence [92,93,94,95].

The biosynthesis of ghrelin, similarly as seen for other gastrointestinal peptide hormones and neuropeptides, is a complex cellular process that involves the initial biosynthesis of a polypeptide precursor that is further cleaved, sorted and post-translationally modified within the regulated secretory pathway [96]. Initially, pre-proghrelin mRNA is translated to 117-residues pre-proghrelin polypeptides, which are synthesized on membrane-bound ribosomes and trans-located into the lumen of the rough endoplasmic reticulum [82]. The N-terminal domain of pre-proghrelin constitutes a 23-residues signal peptide sequence that facilitates its entry to endoplasmic reticulum lumen and is then cleaved by a signal peptidase in order to yield the 94-residues proghrelin polypeptide [82]. The first N-terminal residue in proghrelin is a glycine that constitutes the first residue in the mature ghrelin. Then, proghrelin is subjected to a unique post-translational modification: an *O*-*n*-octanoylation at the serine 3 that is catalyzed by the ghrelin *O*-acyltransferase (GOAT) [66,67]. GOAT belongs to the membrane-bound *O*-acyltransferases family and is highly expressed in ghrelin cells [67]. Both pre-proghrelin and proghrelin seem to be acylated by GOAT since pH for optimal GOAT activity is reached in the endoplasmic reticulum and is lower in further acidic environments of the regulated secretory pathway [97]. Ghrelin is the only known mammalian secreted peptide that presents this fatty acid modification, and it is predicted to be the unique substrate for GOAT within the human proteome [98]. The newly synthesized octanoylated proghrelin is transported to the Golgi complex, from where trafficking pathways emanate in order to generate the mature secretory granules that store bioactive ghrelin. During this vectorial transport, the proghrelin is cleaved. Specifically, a proline-arginine at positions 27–28 of pro-ghrelin is recognized by prohormone convertase 1/3, which cleaves at the C-terminal end of this sequence and generates the mature 28-residues ghrelin [99]. Prohormone convertase 1/3 is expressed in ghrelin cells and it is yet the only identified protease for processing of proghrelin to ghrelin [99,100]. Importantly, the amino acid sequences of mammalian ghrelins is highly conserved, especially in their N-terminal end where the first 10-residues are identical [81,82]. The presence of the octanoyl group at serine 3 is required for the binding of ghrelin to GHSR-1a, and in vitro studies indicated that short peptides containing the first 4 or 5 residues of the N-terminal end of ghrelin are enough to activate GHSR-1a [101,102]. These observations suggest that the N-terminal end of ghrelin constitutes its active core. Notably, proghrelin also gives arise to other molecules in addition to ghrelin. The C-terminal fragment generated by cleavage of proghrelin generates a peptide named obestatin, which was initially reported to display an anorexigenic effect but this was not confirmed in further studies [22,99,100,103,104,105]. Ghrelin is also secreted as a des-octanoylated version, named des-acyl-ghrelin, which circulates in plasma at even higher levels than ghrelin because it is also produced in plasma by ghrelin deacylation [106]. Des-acyl-ghrelin fails to bind to GHSR-1a at physiological concentrations, and it may display some GHSR-1a-independent actions [107]. However, the role of des-acyl-ghrelin as a hormone is also uncertain since no specific receptor for this peptide has been reported, yet [7].

## 3. Ghrelin Synthesis in the Central Nervous System

In 1999, Kojima and colleagues published the seminal study reporting the isolation and identification of ghrelin from rat stomach extracts [51]. The discovery of ghrelin resulted from the search of an endogenous ligand for GHSR-1a. Interestingly, these researchers initially looked for a GHSR-1a ligand in the brain, since this receptor is highly expressed in the hypothalamus and the pituitary; however, the failure to find positive results in over 500 attempts using brain samples turned their attention to other organs [108]. In that groundbreaking article, the authors also reported that pre-proghrelin mRNA was not detected by Northern blot analysis in brain samples, but its presence could be revealed by reverse transcription-polymerase chain reaction (RT-PCR). In addition, they reported the presence of ghrelin-immunoreactive (IR) neurons in the ARC of colchicine-treated rats. It is worth mentioning that Kojima describes in his personal memory that the first version of the article submitted to Nature did not report any central production of ghrelin [108]. Based on the suggestion of one of the reviewers, the authors hypothesized in the revised version of the manuscript that ghrelin could be produced in the hypothalamus. In response to the revised version, the editor requested the inclusion of immunohistochemical (IHC) data supporting the hypothalamic presence of ghrelin. Thus, the second revised version of the article, which was finally accepted, included neuroanatomical data indicating the presence of ghrelin-IR cells in the rat hypothalamus [51].

### 3.1. Presence of Pre-Proghrelin mRNA

After the initial report showing the presence of ghrelin-IR cells in the rat hypothalamus, a number of research groups investigated the putative central ghrelin synthesis. Several studies have documented the presence of pre-proghrelin mRNA in the rat, mouse or human central nervous system using either standard or quantitative RT-PCR (Q-PCR) [51,54,55,57,58,59,60,61,62,65,66,70,71,72,74,75,76,80]. Most studies show that pre-proghrelin mRNA levels in the hypothalamus are much less than in the stomach [54,66,70,72]. However, many times the relative comparisons of mRNA levels cannot be estimated because studies either do not provide stomach mRNA levels or report normalized data of both tissues. Notably, pre-proghrelin mRNA levels are undetectable by in situ hybridization histochemistry (ISHH) or Northern blot analysis in the mouse brain suggesting that the detection of ghrelin gene expression requires amplification steps [51,57,73,79]. Importantly, the first studies that identified GOAT as the critical enzyme for the biosynthesis of ghrelin failed to detect GOAT mRNA in the brain [66,67]. However, subsequent studies using RT-PCR or Q-PCR reported small, but detectable, GOAT mRNA levels in the mouse, rat and human brain [72,74,76,78,80].

### 3.2. Presence of Ghrelin Peptide in Tissue Extracts

Ghrelin peptides have also been detected in extracts from mammalian hypothalamus. The first study in this regard performed separation of peptide extracts from rat hypothalamus using reversed phase high-performance liquid chromatography (RP-HPLC) followed by analysis of ghrelin presence using two different radioimmunoassays (RIAs) [52]. These RIAs were developed using non-commercial polyclonal rabbit antisera against either N-terminal ghrelin (1–11) or C-terminal ghrelin (13–28), and named N-RIA or C-RIA, respectively. This referred study reported that the C-RIA detects the presence of ghrelin-IR signal in rat hypothalamus (~2 fmol/mg tissue), which was ~1000-fold smaller than in the stomach. In contrast, the N-RIA failed to detect ghrelin-IR signal (less than 0.05 fmol/mg tissue) suggesting that des-acyl-ghrelin, rather than the acylated forms of the peptide, was present in the tissue. A similar strategy combining RP-HPLC and N- and C-RIAs was used in other two studies performed by some of the same authors. The first one used the N-RIA and found a rather small ghrelin-IR signal in rat ARC extracts (~0.2 fmol/mg tissue), as compared to the amounts found for other neuropeptides such as GHRH and somatostatin (~20 and 800 fmol/mg tissue, respectively) [58,109]. The second study also detected small amounts of hypothalamic ghrelin (less than 500 fmol per rat hypothalamus) [61]. Notably, this second study reported some inconsistencies, which are not discussed by the authors, in terms of the amount of ghrelin-IR signal detected by N- and C-RIAs in the HPLC fraction corresponding to ghrelin. An independent study investigated the presence of ghrelin peptide in the mouse hypothalamus by also using RP-HPLC combined to two different detection systems and found that ghrelin-IR signal eluting at the position of synthetic ghrelin was undetectable by immunoblotting but unmasked by RIAs commercialized by Phoenix Pharmaceutical [60]. According to the RP-HPLC/RIAs data, ghrelin in the mouse hypothalamus is present in low levels (~30 fmol/mouse, which was ~2500-fold smaller than in the stomach) and represents the major ghrelin-related peptide form, as only low ghrelin-IR signal was detected in the RP-HPLC fraction corresponding to synthetic des-acyl-ghrelin. This study also used two commercial RIAs (from Phoenix Pharmaceuticals and Linco Research), without any separation procedure of the samples nor specifications of the molecules detected by the assays, to measure ghrelin secretion from mouse hypothalamic explants and found ghrelin-IR signal in the incubation medium (~10 fmol/explant). In addition, two independent studies investigated hypothalamic ghrelin secretion in rats [55,75]. One of them used a competitive enzyme immunoassay (EIA) with an antibody against ghrelin (13–28) and showed that ghrelin-IR was present in and secreted from rat hypothalamic explants; unfortunately, the absolute amount ghrelin measured in each condition is unknown because data is reported per assay tube [55]. The other study used a commercial ELISA kit (from Mitsubishi Chemical Medience Corp.) to measure ghrelin secretion from rat hypothalamic explants and showed that ghrelin-IR was present in the secretion medium at low levels (~2 fmol/explant) [75]. Another study used a RIA developed with a polyclonal antibody against ghrelin (1–11), now used in an EIA commercialized by Bertin Pharma, and found that small levels of ghrelin-IR signal are also detected in ewe hypothalamus (~0.2 fmol/mg tissue, less than ~30,000-fold as compared to the abomasum) [40]. The specificity of the assays used to measure ghrelin may be an issue when no separation methods (i.e., HPLC) are performed. When purifications of the samples were not performed, the specificity of the assays in some studies was tested by showing that the amount of ghrelin-IR signal in the samples proportionally decreased with serial dilutions [40,55]. Notably, a more recent study used a commercial ELISA kit (Chemicon), without any separation method, to measure ghrelin levels in peptide extracts from mouse hypothalamus and found that ghrelin-IR signal was present at small levels (~0.1 fmol/mg protein, around ~100-fold less than in the stomach) [77]. Strikingly, this study reported that ghrelin-IR signal in the hypothalamus was similar between ghrelin-knock out (KO) and wild-type mice, while it was dramatically decreased in the stomach and undetectable in the plasma of the former.

### 3.3. Ghrelin Immunohistochemical Staining

The neuroanatomical mapping of ghrelin-IR cells in rodent brains has produced very inconsistent observations. As indicated above, Kojima and colleagues reported that ghrelin-IR neuronal cell bodies were located in the ventrolateral portion of the rat ARC [51]. These IHC studies were performed using a polyclonal rabbit antiserum made against the ghrelin (13–28), which was likely one of the first antiserum raised against ghrelin, and the visualization of ghrelin-IR signal was achieved after amplifications steps using a biotinylated secondary antibody followed by streptavidin-peroxidase and a chromogenic reaction. Then, there is a subset of articles from related research teams that have used a polyclonal rabbit antiserum raised against ghrelin (1–11), and referred as Lot# G606, to study ghrelin neurons in the hypothalamus of non-treated rats or pigs or colchicine-treated rats as well [53,56,58,61,68,69]. This antibody does not bind to des-acyl-ghrelin, but it is unknown if it binds to acylated-ghrelin precursors [52]. All these studies also used an enzymatic amplified chromogenic reaction to visualize ghrelin-IR signal. The specificity of the immunostaining was tested by incubation of brain sections with a pre-immune serum, which showed no signal. Light microscopy analysis showed the presence of ghrelin-IR cell bodies and fibers of oval/polygonal neurons located in the ventral portion of the ARC. Electron microscopy revealed that ghrelin-IR signal was present in almost all cell organelles, except the nucleus, the mitochondria and the multiple vesicular bodies, with the ghrelin-IR signal being more specifically located in the Golgi apparatus and dense granular vesicles. The authors stressed the observation that ghrelin-IR signal by electron microscopy was not detected when glutaraldehyde was used as a fixative; thus, brains were fixed with 4% paraformaldehyde and, as a consequence, the ultrastructure was poorly preserved [53]. Another set of neuroanatomical analysis was performed using three independent non-commercial affinity-purified polyclonal antibodies raised against an unspecified part of ghrelin that recognize a band of 13 kDa that matches the estimated size of proghrelin [54]. The specificity of the assay was tested by pre-adsorption of the antisera with ghrelin and by showing that brain sections of ghrelin-KO mice lacked ghrelin-IR signal. It was not reported if the antibody also recognizes des-acyl-ghrelin. Immunostainings were performed in the hypothalamus of naïve and colchicine-treated rats, and the visualization of the ghrelin-IR signal was also performed using an enzymatically amplified chromogenic reaction. This study reported the presence of ghrelin-IR in the cell bodies and fibers of bipolar neurons that were located in the hypothalamic space between the lateral hypothalamus (LH), the ARC, dorsomedial nucleus (DMN), ventromedial nucleus (VMN), the paraventricular nucleus (PVN) and the ependymal layer of the third ventricle. Electron microscopy analysis showed that ghrelin-IR signal was present in dense-core vesicles within neuronal terminals. Another study used one of these three antibodies and the same immunostaining procedure to investigate the presence of ghrelin in the human brain, and showed the presence ghrelin-IR fibers, but not cell bodies, in the suprachiasmatic nucleus (SON), the periventricular nucleus (Pev), the PVN, the DMN, the VMN, the perifornical region and the infundibular nucleus [64]. An independent team studied the localization of ghrelin-IR cells in the rat brain using a goat antiserum against the internal region of ghrelin commercialized by Santa Cruz Biotechnology (Cat# SC-10368, [62]). Here, ghrelin-IR signal was visualized using a fluorescent anti-goat antibody, and control studies were performed by showing the lack of ghrelin-IR signal when the primary antibody was omitted or replaced by normal rabbit serum. This study reported that ghrelin-IR cells in the rat were located in the LH, the PVN, the ARC and the ependymal layer of the third ventricle as well as in the sensorimotor cortex and cingulate gyrus. A different team used an undefined polyclonal rabbit antibody against an unidentified portion of ghrelin to perform chromogenic IHC in rat hypothalamus and reported the presence of ghrelin-IR cell bodies in the ARC [63]. More recently, another study reported the use of three non-commercial anti-ghrelin antibodies, which recognize the N-terminal portion of the molecule, to test the presence of ghrelin-IR cells in rodent brains [77]. In this study, ghrelin-IR visualization was achieved by a fluorescent secondary antibody, an enzymatically amplified chromogenic reaction, or an ultrasensitive commercial system—the MACH 4 Universal HRP-Polymer. Under these conditions, ghrelin-IR cell bodies were detected scattered throughout the rat and mouse hypothalamus, being more prominently located in the ARC, the SON and around the third ventricle; however, the ghrelin-IR signal was not reduced by absorbing concentrations of ghrelin peptide and it was still present in ghrelin-KO mice [77].

### 3.4. Ghrelin Knock out and Ghrelin-Gene Reporter Mice

The study of genetically manipulated mouse models has been critical for the understanding of the mechanisms underlying ghrelin’s effects [110], and has also provided additional evidences regarding the potential biosynthesis of ghrelin in the brain. A first ghrelin-KO mouse model was generated by replacing the entire coding region of ghrelin gene by the bacterial β-galactosidase gene through homologous recombination [57]. In order to validate the gene deletion, immunostaining against ghrelin was performed using a commercial rabbit antiserum that recognizes pre-proghrelin and cannot distinguish between ghrelin and des-acyl-ghrelin (Phoenix Pharmaceuticals, Cat# H-191-031-31). Immunostaining showed that ghrelin-KO mice lacked ghrelin-IR cells in the stomach and small intestine, but showed lightly stained ghrelin-IR cells in the hypothalamus with a similar pattern as seen in wild-type mice. Furthermore, β-galactosidase positive cells were not detected in the hypothalamus of ghrelin-KO mice, despite they were present in the stomach [57].

In order to study ghrelin-producing cells, some transgenic reporter mouse lines were also generated [70,73]. In one of these transgenic models, the enhanced green fluorescent protein (EGFP) was expressed under the control of an 8.6 kb DNA fragment containing transcription regulatory regions of the mouse ghrelin gene [70]. These ghrelin-EGFP mice showed ghrelin and EGFP mRNA in both the stomach and the hypothalamus. However double EGFP-IR and ghrelin-IR cells were seen in the stomach but not in the hypothalamus, under the same experimental conditions. Only the use of wide-band spectral confocal laser microscopy in brain sections of these transgenic mice could unmask the presence of faint fluorescent cells in the ARC, but the specificity of this signal or its intensity as compared to wild-type mice was not reported [70]. Other transgenic reporter mouse lines expressed the humanized *Renilla reniformis* GFP (hrGFP) driven by mouse ghrelin gene regulatory elements [73]. In this case, constructs were generated using two different ghrelin bacterial artificial chromosomes, which contained different amounts of genomic sequence flanking the ghrelin gene. One construct contained ~192.0 kb upstream of the ghrelin start codon and ~1.2 kb downstream of the ghrelin stop codon, while the other contained ~59.4 kb upstream of the start codon and ~103.7 kb downstream of the stop codon. In both constructs, the initial portion of ghrelin coding sequence was replaced by hrGFP. Ghrelin-hrGFP mice strains generated with each of these two constructs showed abundant hrGFP fluorescent cells, which co-localized with ghrelin-IR signal, in the stomach but hrGFP fluorescent or hrGFP-IR cells could not be detected in the hypothalamus. In the same study, immunostaining against ghrelin using the same two commercial antibodies from Phoenix Pharmaceutical or from Santa Cruz Biotechnology referred above, and ISHH for ghrelin mRNA failed to give any positive signal in the brains of these transgenic mice or wild-type mice [73].

## 4. Discussion

The fact that the detection of ghrelin gene expression in mammalian brain requires highly sensitive PCR methods while other less sensitive approaches—Northern blot analysis or ISHH—fail to detect it indicates that pre-proghrelin mRNA is, at best, present at very low levels in normal conditions. This is in contrast to most neuropeptide mRNAs, which are robustly expressed in the brain and can be easily detected by techniques such as ISHH. It should be also kept in mind that the detection of mRNA transcripts containing some sequences derived from the ghrelin gene does not necessary mean that the pre-proghrelin mRNA is expressed in the brain due to the complex transcriptional pattern of this gene. Indeed, the detection of different mRNA transcript variants derived from the ghrelin gene may be one of the underlying reasons for the inconsistencies found among different techniques and/or different studies looking for ghrelin gene expression in the brain. Moreover, observations based on mouse models with manipulations of the ghrelin gene also suggest that the ghrelin gene promoter is not significantly active in the mouse brain. In addition, it is important to stress that the synthesis of bioactive ghrelin requires the enzymatic action of GOAT. However, it is unclear if GOAT gene is centrally expressed as many studies failed to detect its transcript in the brain, even using RT-PCR. Such low levels of expression for both ghrelin and GOAT genes not only make very difficult to experimentally address essential aspects of this system (i.e., whether hypothalamic pre-proghrelin mRNA is translated to protein; whether both pre-proghrelin and GOAT mRNAs are present in the same cell; etc.) but also raise concerns in term of its physiological implications.

The use of antibody-based detection methods have provided data indicating that ghrelin-IR signal exists in the brain, and several authors used these observations as indirect evidences that ghrelin gene-derived peptides can be synthesized in the central nervous system. Unfortunately, most of the studies reporting ghrelin-IR signal in the brain were performed using non-commercial antibodies; thus, the ability of other laboratories to confirm such observations has been limited. To our knowledge, only three studies have used commercially available anti-ghrelin antibodies to look for ghrelin-IR cells in the rodent´s brain. Two independent studies failed to detect ghrelin-IR signal in the mouse brain using the same anti-ghrelin antibody commercialized by Phoenix Pharmaceutical [57,73]. One study reported the visualization of ghrelin-IR neurons in the rat brain using an antibody produced by Santa Cruz Biotechnology [62], but the distribution of ghrelin-IR neurons shown in that study could not be confirmed in either mice (using the same antibody, [73]) or rats (using non-commercial anti-ghrelin antibodies, [77]). The use of different anti-ghrelin antibodies is likely one of the main reasons for the discrepancies in term of the localization of ghrelin-IR neurons in the central nervous system. Importantly, most studies have accurately used stomach samples as a positive control to confirm that anti-ghrelin antisera (or antibodies) can label ghrelin-producing cells. However, it is not always clear if the anti-ghrelin antisera (or antibodies) used in the different studies can also bind to other molecules. Some studies report that the labeling of the brain sections was blocked by pre-incubating the antiserum (or antibody) with ghrelin as specificity control; however, this procedure only demonstrates that the antiserum (or antibody) is specific for ghrelin but it does not test if the antibody binds to the same or other molecules in the tissue [111]. Similarly, the absence of immunostaining when the primary antibody is omitted or replaced by pre-immune antiserum is a control for nonspecific binding of the secondary antibody, but does not evidence anything in terms of the specificity of the immunostaining against ghrelin [111]. The ultimate trustworthy specificity control of an anti-ghrelin antibody is the lack of immunostaining in samples of ghrelin-KO mice, in which ghrelin must be absent [112]. As referred above, only three reports have performed this negative control assay to test the specificity of the used anti-ghrelin antibodies [54,57,77], and only one out of three found differential labeling between brain sections of wild-type vs. ghrelin-KO mice [54]. Indeed, one of the studies found similar neuron-shape ghrelin-IR signal in both type of mice suggesting that some anti-ghrelin antibodies can bind to some ghrelin-mimicking substances in brain tissue [77]; such findings raise concerns of the outcomes of other studies that have not performed such test of specificity. On the other hand, an accurate interpretation of neuroanatomical data requires the precise knowledge of all the ghrelin-related molecules recognized by a given anti-ghrelin antibody. Assuming that neurons produce ghrelin and that its biosynthesis occurs in the same way as shown in ghrelin-producing cells of the stomach, proghrelin precursor should be mostly present in the endoplasmic reticulum and the Golgi apparatus surrounding the cellular nucleus, while the ghrelin peptide would mainly be present in immature and mature secretory granules distributed thought the neuronal terminals and axons. Thus, immunostaining of brain sections of naïve animals using antibodies that recognize proghrelin and/or ghrelin should label the cell body and/or the terminals of the neurons, respectively, depending on the specificity and selectivity of the antibodies. In brain sections of animals treated with colchicine, both types of antibodies should mainly label neuronal cell bodies, as colchicine disrupts the axonal transport and induces the accumulation of neuropeptides in the soma [113]. As seen in Table 1, reported immunohistochemical observations do not always follow these predictions, and the incomplete description of the ghrelin-related molecules recognized by some anti-ghrelin antibodies makes it hard to understand the causes of such outcomes. In addition, none of the studies described above displayed comparative images of ghrelin-IR signal in the hypothalamus of naïve versus colchicine-treated animals. Data demonstrating the accumulation of signal in the neuronal cell bodies of the hypothalamus of colchicine-treated animals would provide a fair support of the notion that ghrelin may be locally produced.

The use of a separation method, such as RP-HPLC, followed by the quantification of ghrelin-IR signal using two independent assays against N-terminal and C-terminal epitopes of the peptide strongly indicated that intact ghrelin can be detected in the hypothalamus, although at very low levels as compared to the stomach. However, such an experimental strategy does not provide information to evidence if the detected ghrelin is either synthetized in situ and located inside the neurons, or coming from plasma and located in the extracellular compartment or internalized through GHSR-1a-mediated mechanisms. Assuming that peptide extracts from the hypothalamus lack any cerebrospinal fluid or plasma contamination that could be the source of ghrelin, it is still plausible that the ghrelin detected in the brain tissue is circulating hormone that have accessed the hypothalamic parenchyma. Notably, many studies have reported the presence of ghrelin-IR signal in the ARC, which highly expresses GHSR-1a and is located in close apposition to the ME. The ME is a key circumventricular organ in which circulating ghrelin passively extravasates to the nearby ARC to, in turn, bind to GHSR-1a-expressing neurons [12,13,37]. Thus, it cannot be ruled out that ghrelin-IR signal detected in the ARC represents stomach-produced peptide that has reached the hypothalamus from bloodstream. Unfortunately, none of the reports investigating the localization of ghrelin-IR neurons have used high resolution fluorescent microscopy in order to gain some insights of the specific localization of the labeling. Electron microscopy analyses have shown that ghrelin-IR signal is present in dense-cored vesicles of axon terminals. However, it is known that GHSR-1a is located at presynaptic terminals [26,54], and that ghrelin/GHSR-1a complexes are progressively internalized after the binding of ghrelin and accumulated intracellularly for around three hours [114]. Thus, the source of the ghrelin detected in these studies remains unclear.

## 5. Conclusions

Based on the data reviewed here in and summarized in Table 2, we conclude that no irrefutable and reproducible evidence exists supporting the notion that ghrelin is synthetized, at physiologically relevant levels, in the central nervous system of adult mammals. Thus, we propose that the notion that ghrelin is produced in the brain has been unjustifiably installed in part of the scientific community due to a variety of reasons, including the fact that the initial studies showing positive indications of ghrelin presence in the brain were published in high-impact journals while further evidences failing to support these initial observations were not published in such highly visible journals or published as a supplementary part of other studies (i.e., in the validation of genetically-modified mice). In addition, the difficulties in publishing confident negative data together with the well-established difficulties in science to correct existing ideas may have contributed to perpetuate the idea that neurons can synthetize ghrelin. We hope the current review will help the scientific community to adopt a more objective point of view of this topic, which is that a careful reading of the literature indicates that ghrelin is not produced at significant amounts in the brain.

## Figures and Tables

**Table 1:** Studies reporting pre-proghrelin mRNA or ghrelin peptide in in central nervous system of mice, rats or humans.

First Author-Date	Experimental Strategy	Main Observations	Reference
Kojima-1999	Northern blot using a 620 bp probe against pre-proghrelin mRNA in rat brain.	No signal.	[51]
	RT-PCR using primers that amplify exon 1 to 4 sequence of pre-proghrelin mRNA in rat brain.	Positive gene expression.	
	IHC using a non-commercial rabbit polyclonal antibody against C-terminal ghrelin (13–28) in colchicine-treated rat hypothalamus.	Ghrelin-IR cell bodies in the ARC.	
Hosoda-2000	RP-HPLC separation of peptide extracts from rat hypothalamus followed by two RIAs, named N-RIA and C-RIA, developed using non-commercial rabbit polyclonal antisera against either N-terminal ghrelin (1–11) (#G606 antiserum) or C-terminal ghrelin (13–28) (#G107 antiserum), respectively.	Ghrelin-IR signal in hypothalamus, which was ~1000-times smaller than in the stomach, using C-RIA. In contrast to the stomach, no signal was detected in the hypothalamus using the N-RIA.	[52]
Lu-2002	IHC using #G606 antiserum against ghrelin (1–11) in colchicine-treated rat hypothalamus.	Ghrelin-IR cell bodies and their terminals in the ARC. Ghrelin-IR signal was detected in the Golgi apparatus and dense granular vesicles by electron microscopy.	[53]
Cowley-2003	RT-PCR in mouse and rat hypothalamus using primers against pre-proghrelin mRNA, as used by Kojima et al. 1999.	Positive gene expression.	[54]
	Western blot using a non-commercial affinity-purified polyclonal antisera against an unspecified part of ghrelin in protein extracts from rat hypothalamus.	A band of 13 kDa corresponding to the estimated proghrelin mass.	
	IHC using same antibodies used for western blot in naïve and colchicine-treated rat as well as mouse hypothalamus.	Ghrelin-IR cell bodies located in the hypothalamic space between the LH, ARC, DMN, VMN, PVN and ependymal layer of the third ventricle. No labeling was detected in sections of the ghrelin-KO mice. Ghrelin-IR signal was also detected by electron microscopy in neuronal terminals, and located in dense-cored vesicles.	
Mozid-2003	RT-PCR using primers that amplify exons 1 to 3 sequence of pre-proghrelin mRNA in rat hypothalamus..	Positive gene expression.	[55]
	Competitive EIA using a rabbit polyclonal antibody against ghrelin (13–28) in rat hypothalamic homogenates and secretion media.	Ghrelin-IR signal.	
Guan-2003	IHC using #G606 antiserum against ghrelin (1–11) in rat hypothalamus.	Ghrelin-IR cell bodies and their terminals in the ARC. Ghrelin-IR signal was detected in dense granular vesicles by electron microscopy.	[56]
Wortley-2004	Northern blot using a full length ghrelin probe in mouse hypothalamus.	No signal.	[57]
	Q-PCR (TaqMan) using an unspecified set of primers against pre-proghrelin mRNA in mouse hypothalamus.	Positive gene expression.	
	IHC using a rabbit serum against rat octanoylated ghrelin (Phoenix Pharmaceuticals, Cat# H-191-031-31) in mouse brain.	No signal.	
Mondal-2005	RT-PCR in rat ARC using primers against pre-proghrelin mRNA, as used by Kojima et al. 1999.	Positive gene expression.	[58]
	IHC using #G606 antiserum against ghrelin (1–11) in colchicine-treated rat ARC.	Ghrelin-IR cell bodies.	
	RP-HPLC separation of peptide extracts from rat ARC followed by a RIA using the same antibody than for IHC.	A peak that elutes at the position of ghrelin.	
Turek-2005	Q-PCR using an unspecified set of primers against pre-proghrelin mRNA in mouse hypothalamus.	Positive gene expression.	[59]
Hu-2005	RT-PCR using an unspecified set of primers against pre-proghrelin mRNA in hypothalamic mouse explants.	Positive gene expression.	[60]
	RP-HPLC separation of peptide extracts from mouse hypothalamus followed by commercial RIAs (Phoenix Pharmaceuticals) to measure ghrelin or ghrelin plus des-acyl-ghrelin. Some HPLC fractions were subjected to SDS/PAGE and immunoblotting using an unspecified anti-ghrelin antibody from Phoenix Pharmaceuticals.	Immunoblotting shows no ghrelin-IR signal, while RIAs of HPLC fractions detect ghrelin-IR signal that elutes at the position of ghrelin and accounts the majority of the total ghrelin-IR signal.	
	RIA analysis of ghrelin content in and secreted from mouse hypothalamic explants using commercial kits form Phoenix Pharmaceuticals and Linco Research.	Ghrelin-IR signal in secretion medium.	
Sato-2005	RT-PCR using primers that amplify exons 2 to 3 sequence of pre-proghrelin mRNA in rat hypothalamus.	Positive gene expression.	[61]
	IHC #G606 antiserum against ghrelin (1–11) in porcine hypothalamus.	Ghrelin-IR in the ARC, the PVN and periventricular regions.	
	RP-HPLC separation of peptide extracts from rat hypothalamus followed by N-RIA and C-RIA, as described in Hosoda et al. 2000.	A peak that elutes at the position of ghrelin was detected by both N-RIA and C-RIA.	
Hou-2006	RT-PCR in rat hypothalamus and frontal cortex using primers against pre-proghrelin mRNA, as used by Kojima et al. 1999.	Positive gene expression.	[62]
	IHC using a goat antiserum against ghrelin (Santa Cruz Biotechnology, Cat# sc-10368) in rat hypothalamus and frontal cortex.	Ghrelin-IR cells in the LH, the PVN, the ARC and the ependymal layer of the third ventricle. In cerebral cortex, Ghrelin-IR cells mainly distributed in sensorimotor cortex and cingulate gyrus.	
Canpolat-2006	IHC using an unspecified rabbit polyclonal antibody in rat hypothalamus.	Ghrelin-IR cell bodies in the ARC.	[63]
Menyhert-2006	IHC in human hypothalamus using the non-commercial antibody, as used by Cowley et al. 2003.	Ghrelin-IR fibers, but not cell bodies in the SON, Pe, SQN, PVN, perifornical, infundibular nucleus, DMN and VMN.	[64]
Luque-2007	Q-PCR using primers that amplify exons 2 to 3 sequence of pre-proghrelin mRNA in mouse hypothalamus.	Positive gene expression.	[65]
Yang-2008	RT-PCR using sets of primers that amplify sequences from exons 1 to 4 of pre-proghrelin or GOAT mRNAs in mouse brain.	Negative gene expression of GOAT and very little expression of ghrelin.	[66]
Gutierrez-2008	TissueScan Real-Time with human cDNA panels (Origene Technologies) for pre-proghrelin and GOAT mRNAs. Primer sequences are not provided.	Negative gene expression for both genes in the brain.	[67]
Guan-2008	IHC using the #G606 antiserum and a goat antiserum both against anti-ghrelin (1–11) in rat hypothalamus.	Ghrelin-IR cell bodies and their terminals in the ARC. Ghrelin-IR signal was detected in dense granular vesicles by electron microscopy.	[68]
Hori-2008	IHC using #G606 and #G4-2 non-commercial rabbit polyclonal antisera against ghrelin (1–11) in rat hypothalamus.	Ghrelin-IR cell bodies and their terminals in the ARC. Ghrelin-IR signal was detected in dense granular vesicles by electron microscopy.	[69]
Kageyama-2008	RT-PCR using primers that amplify exons 2 to 4 of pre-proghrelin mRNA in mouse hypothalamus.	Positive gene expression.	[70]
	Ghrelin-EGFP transgenic mouse model, in which EGFP gene expression is driven by ghrelin promoter.	EGFP-IR cells in the ARC only detected by wide-band spectral confocal laser microscopy but not observed by conventional fluorescence microscopy.	
Grouselle-2008	Competitive EIA using a rabbit polyclonal antibody against ghrelin (1–11) in sheep hypothalamic homogenates.	Ghrelin-IR signal in hypothalamus, which was ~30,000-fold smaller than in abomasums and ~1000-fold smaller than in small intestine.	[40]
Ueberberg-2009	Q-PCR using primers that amplify exon 1 sequence of pre-proghrelin mRNA in human brain.	Positive gene expression.	[71]
Sakata-2009	-RT-PCR using sets of primers that amplify sequences from exons 1 to 2 of pre-proghrelin or GOAT mRNAs in mouse brain.	Positive gene expression for both genes.	[72]
Sakata-2009	Ghrelin-hrGFP transgenic mouse models in which hrGFP gene expression is driven by ghrelin promoter.	No signal of hrGFP fluorescence or hrGFP-IR in the brain.	[73]
	IHC using two different commercial rabbit anti-ghrelin antisera (Phoenix Pharmaceuticals, Cat# H-191-031-31 or Santa Cruz Biotechnology, SC-10368) in ghrelin-hrGFP transgenic or wild-type mice brains.	No signal.	
	ISHH targeting exon 4 sequence of pre-proghrelin mRNA in brains of ghrelin-hrGFP transgenic or wild-type mice.	No signal.	
Gahete-2010	Q-PCR using sets of primers that amplify sequences from exon 2 to 3 of pre-proghrelin or GOAT mRNAs in mouse hypothalamus.	Positive gene expression for both genes.	[74]
Yakabi-2010	Q-PCR (TaqMan) using primers that amplify sequences from exon 2 to 3 of pre-proghrelin mRNA in rat hypothalamus.	Positive gene expression.	[75]
	ELISA (Mitsubishi Chemical Medience Corp.) against ghrelin in rat hypothalamic homogenates and secretion media.	Positive ghrelin-IR signal in content and secretion.	
Gahete-2010	Q-PCR using sets of primers that amplify sequences from exon 1 to 2 of pre-proghrelin or GOAT mRNAs in human temporal lobe.	Positive gene expression for both genes.	[76]
Furness-2011	IHC in rat and mouse brains using different non-commercial rabbit polyclonal antisera: (1) RY1601, against rat ghrelin (1–15)-Cys coupled to keyhole limpet hemocyanin; (2) RY1595, against rat des-acyl-ghrelin (1–15)-Cys coupled to keyhole limpet hemocyanin; (3) GO-1, against human ghrelin coupled through glutaraldehyde to bovine serum albumin; (4) GM-2, against human ghrelin coupled through carbodiimide to bovine serum albumin.	No specific signal.	[77]
	ELISA (Chemicon) against ghrelin in rat and mouse hypothalamus.	Very low levels of ghrelin.	
Rucinski-2012	RT-PCR and Q-PCR for GOAT mRNA in rat hypothalamus.	Positive gene expression.	[78]
Francois-2015	ISHH targeting exons 1 to 3 sequences of pre-proghrelin mRNA in mouse hypothalamus.	No signal.	[79]
Wellman-2015	Q-PCR using primers that amplify exons 1 to 4 sequence of pre-proghrelin and GOAT mRNAs in rat hypothalamus.	Positive gene expression for both genes.	[80]

ARC, arcuate nucleus; IR, immunoreactive; IHC, immunohistochemical; RIA, radioimmunoassays; EIA, enzyme immunoassay; GOAT, ghrelin *O*-acyltransferase; EGFP, enhanced green fluorescent protein; LH, lateral hypothalamus; DMN, dorsomedial nucleus; VMN, ventromedial nucleus; PVN, paraventricular nucleus; SON, supraoptic nucleus; SQN, suprachiasmatic nucleus.

**Table 2:** Summary of the arguments in favor or against a local synthesis of ghrelin in brain.

Level	Experimental Findings	Main Conclusions
pre-proghrelin mRNA	No signal by Northern blot or ISHH. Positive signal by RT-PCR or Q-PCR.	Pre-proghrelin mRNA measurable in low amounts
Ghrelin peptide	Presence of ghrelin in amounts less than 100- to 5000-fold as compared to stomach levels	Ghrelin measurable in minute amounts but no conclusive evidence for local synthesis.
Cellular localization.	Presence of ghrelin-IR positive neurons in many nuclei and regions	No reproducibility amongst studies. Lack of adequate specificity controls, namely absence in ghrelin KO mice. Cellular location of the positive signal unexpected, based on the epitope recognized by the antibodies.
